# Treatment Strategies for Painful Pelvic Floor Conditions: A Focus on the Potential Benefits of Cannabidiol

**DOI:** 10.3390/biom14121627

**Published:** 2024-12-19

**Authors:** Roberto Bonanni, Patrizia Ratano, Ida Cariati, Virginia Tancredi, Pierangelo Cifelli

**Affiliations:** 1 Department of Biomedicine and Prevention, “Tor Vergata” University of Rome, Via Montpellier 1, 00133 Rome, Italy; roberto.bonanni1288@gmail.com; 2 Department of Food Safety, Nutrition and Veterinary Public Health, Istituto Superiore di Sanità (ISS), 00161 Rome, Italy; patrizia.ratano@iss.it; 3 Department of Systems Medicine, “Tor Vergata” University of Rome, Via Montpellier 1, 00133 Rome, Italy; ida.cariati@uniroma2.it; 4 Centre of Space Bio-Medicine, “Tor Vergata” University of Rome, Via Montpellier 1, 00133 Rome, Italy; 5 Department of Applied Clinical and Biotechnological Sciences, University of L’Aquila, 67100 L’Aquila, Italy; pierangelo.cifelli@univaq.it

**Keywords:** pelvic floor conditions, cannabidiol, endocannabinoid system, cannabinoid receptors, physiology, pelvic pain

## Abstract

Painful conditions of the pelvic floor include a set of disorders of the pelvic region, discreetly prevalent in the female population, in which pain emerges as the predominant symptom. Such disorders have a significant impact on quality of life as they impair couple relationships and promote states of anxiety and irascibility in affected individuals. Although numerous treatment approaches have been proposed for the management of such disorders, there is a need to identify strategies to promote muscle relaxation, counter pelvic pain, and reduce inflammation. The endocannabinoid system (ECS) represents a complex system spread throughout the body and is involved in the regulation of numerous physiological processes representing a potential therapeutic target for mood and anxiety disorders as well as pain management. Cannabidiol (CBD), acting on the ECS, can promote relief from hyperalgesia and allodynia typical of disorders affecting the pelvic floor and promote muscle relaxation by restoring balance to this delicate anatomical region. However, its use is currently limited due to a lack of evidence supporting its efficacy and harmlessness, and the mechanism of action on the ECS remains partially unexplored to this day. This comprehensive review of the literature examines the impact of pain disorders affecting the pelvic floor and major treatment approaches and brings together the main evidence supporting CBD in the management of such disorders.

## 1. Introduction

The pelvic floor is a rhomboid region extending from the pubic symphysis to the coccyx and including ligaments, muscles, and fascia that collectively provide support to the bladder, reproductive organs, and rectum [1]. This supportive function relies on the anatomical relationships established by the soft tissues of the pelvic floor with the surrounding bony, joint, and muscular structures, such as the pelvic girdle, sacroiliac joints, pubic symphysis, as well as hip and gluteal muscles [2]. From a physiological perspective, the pelvic floor performs multiple functions as it is involved in urination, reproduction and defecation [3]. Thus, several conditions characterized by the onset and persistence of pelvic pain can induce urinary, vaginal, or anorectal symptoms [4]. From a clinical point of view, these disorders can be identified as urinary incontinence, pelvic organ prolapse, fecal incontinence, or painful syndrome of the pelvic-perineal region [5,6,7]. Data on the prevalence of painful disorders of the pelvic floor are highly variable and can be as high as 25% in healthy non-pregnant United States women [5,8] and up to 46.5% in Japanese adult women [8,9].

According to a report published by Peinado-Molina and colleagues in 2023, out of 1456 women participating in an observational study conducted in Spain, 55.8% suffered from urinary incontinence, 10.4% from fecal incontinence, 14% from symptomatic uterine prolapse, and 18.7% from pelvic pain, highlighting the pandemic dimensions of these problems [10]. Importantly, it has been reported that the estimated risk of painful disorder of pelvic floor increases considerably in women who participate in sports, highlighting the major limitations that such conditions place on the careers of those affected in addition to their daily lives [11]. Furthermore, it should be considered that painful pelvic floor disorders’ prevalence data do not reflect the true number of women involved, as many cases remain undiagnosed due to the social, emotional, and psychological impact that these conditions entail [12]. Therefore, identification and development of strategies aimed at counteracting the conditions characterized by pelvic pain must be a primary goal of medical research in the field. In this context, physical therapy aimed at strengthening the pelvic floor muscles is an excellent tool for the functional retraining of this anatomical area [13]. However, in some cases, such as endometriosis and vulvodynia, it is necessary to resort to pharmacological treatments, with results that are often weak or associated with side effects [14,15]. In this context, the analgesic and anti-inflammatory properties of cannabidiol (CBD) could provide a valuable aid to the treatment of patients with painful disorders of the pelvic floor, as it has been reported that the cannabinoid can produce positive results in the treatment of dysmenorrhea, dyspareunia, vaginismus, and pudendal neuropathy [16,17]. Based on this evidence, the purpose of this review is to gather evidence regarding the effects of CBD on pelvic floor pain and pathology to evaluate CBD as a potential additional treatment strategy for patients with painful pelvic floor disorders.

## 2. Painful Pelvic Floor Conditions

Painful pelvic floors conditions include a wide variety of diseases and symptoms in which there are anatomical changes related to abnormal function of the pelvic floor muscles. In general, these disorders can arise because of increase, decrease, or abnormal coordination of the pelvic floor muscle activity, leading to the appearance of urological, gynecological, or colorectal symptoms. To date, the underlying causes of the onset of pelvic floor pain disorders have not yet been fully identified, as no specific or trigger event has been identified. On the other hand, several factors have been associated with an increased risk of pelvic pain occurrence, such as chronic avoidance of urination and bowel movements [18,19], posture and gait asymmetry [20,21], inflammatory diseases [22], spinal nerve injuries [23], as well as prostatectomy surgery [24].

### 2.1. Dysmenorrhea

Dysmenorrhea, characterized by severe and frequent menstrual cramps and pain during periods is a disorder with an enormous social impact as it affects 50% to 90% of adolescent girls and women of reproductive age [25]. Alongside the presence of other pelvic floor diseases, such as endometriosis or infection, dysmenorrhea can result in progressively worsening algic symptoms, abnormal uterine bleeding, vaginal discharge, and dyspareunia [25]. Despite the widespread occurrence of dysmenorrhea and significant research efforts in this field, the pathogenetic mechanism underlying this condition remains unclear. It has been suggested that increased concentrations of prostaglandins, in particular prostaglandin F2α (PGF2α) and prostaglandin E2 (PGE2), prior to menstruation might contribute to the development of dysmenorrhea by leading to the narrowing of vessels supplying blood to the uterus and the alteration of normal contractile activity, promoting ischemia, hypoxia, and increased sensitivity of uterine nerve endings [26]. It is noteworthy that although a role of vasopressin and oxytocin in the onset of dysmenorrhea has been suggested [27], conflicting results have been reported on the use of receptor antagonists of these hormones [28,29], highlighting the need to identify additional strategies that can counteract this condition.

### 2.2. Vaginismus and Dyspareunia

Vaginismus, with an estimated prevalence between 0.5 and 1 percent of the female population [30], depends on painful involuntary spasms of the vaginal muscles that make it difficult or impossible to participate in sexual intercourse [31]. Although pain is often identified as the predominant symptom of vaginismus, it is unclear whether hyperalgesia is the cause or consequence of vaginal muscle spasm [32]. Similarly, dyspareunia is a condition characterized by persistent or recurrent genital pain felt specifically during sexual intercourse. The World Health Organization reported a prevalence of between 8% and 21.1%, but highly variable estimates have been reported depending on the geographical area considered [33]. Dyspareunia can be classified as superficial or deep, depending on pain location, and can be due to pelvic region disorders, such as vulvodynia or endometriosis [34]. It is noteworthy that vaginismus can often be secondary to dyspareunia, making the boundary between the two conditions extremely blurred [35]. For this reason, in the fifth edition of the Diagnostic and Statistical Manual of Mental Disorders (DSM-5), vaginismus and dyspareunia have been integrated into the same diagnostic category, termed genito-pelvic pain/penetration disorder (GPPPD) [36]. Despite the impact of these conditions on the private lives of affected women, the effectiveness of treatments tested to date is quite variable, as it is dependent on the emotional and cognitive state of the individual, as well as on the pain-associated behaviors.

### 2.3. Pudendal Nerve Neuralgia

Pudendal nerve neuralgia is a chronic, often severely disabling neuropathic pain condition induced by inflammation, compression, or entrapment of the pudendal nerve [37]. This results in the onset of pain, constant or intermittent, which can be described as burning, tingling, or stabbing pain in the pelvic, perineal, or genital area. The International Pudendal Neuropathy Association estimates an incidence of 1 in 100,000 in the general population, although European estimates are around 4% [38]. This condition remains significantly underdiagnosed or, in many cases, misdiagnosed, making it difficult to estimate the prevalence within the population [38]. Compression of the pudendum, referred to as “entrapment,” is one of the most common mechanical causes of pudendal neuralgia and is often caused by spasm of pelvic floor muscles, pressure from surrounding ligaments, or scar tissue from trauma or surgery [39,40]. Alternatively, a previous surgical procedure for prolapse or incontinence may also result in pudendal entrapment caused by a mesh or suture [41]. In compression injuries of the pudendal, the blood supply to the nerve is compromised, inducing demyelination that results in functional disturbance that may include mild paresthesia, motor weakness, and muscle paralysis [42]. As an alternative to compression, other causes of nerve damage can be stretches, commonly related to childbirth, and transposition injuries [43]. Finally, herpes simplex infection and inflammation caused by neoplasms, chemotherapy, and endometriosis may also be other causes of pudendal neuralgia [43,44].

### 2.4. Perineal Trauma

Perineal trauma includes any type of damage to the female genitalia as a complication of vaginal birth and can be distinguished into anterior, when it involves the anterior vaginal wall, urethra, clitoris, and labia, or posterior when the posterior vaginal wall, perineal muscle, perineal body, external and internal anal sphincters, and anal canal are involved [45]. During labor, most perineal injuries occur along the posterior vaginal wall, extending toward the anus [45]. Perineal trauma at the time of vaginal delivery is common, and when the anal sphincter is included, injuries may be associated with additional morbidity including incontinence, pelvic pain, and sexual dysfunctions [46]. The estimated incidence of postpartum perineal trauma is about 0.1%, highlighting the important dimensions of this problem [47]. In fact, the economic burden associated with this type of complication ranges between GBP 3.7 and 9.8 million in the United Kingdom, in cases of assisted or spontaneous vaginal delivery, respectively, while it has reached USD 83 million in the United States [48]. Several authors have reported evidence on the effectiveness of pre-partum perineal messaging [49], but the management of the postpartum perineal trauma, as well as related consequences, remains open.

### 2.5. Chronic Constipation

Chronic constipation is a gastrointestinal disorder, characterized by difficult and/or infrequent passage of feces in the colon, with a prevalence in the general population of 20%, affecting mostly women, and significantly affecting quality of life [50]. Indeed, the consequences of chronic constipation include excessive straining, incomplete evacuation feeling, failed or prolonged defecate attempts, use of digital maneuvers to facilitate stool evacuation, abdominal bloating, and hard stool consistency. Usually, difficulties in stool evacuation may be due to either insufficient relaxation of the pelvic floor muscles or the inability to generate adequate propulsive forces during defecation [51].

### 2.6. Endometriosis

Endometriosis is a condition characterized by a chronic inflammatory process in the endometrium that affects 5 to 10% of women of reproductive age globally and results in chronic pelvic pain, dysmenorrhea, dyspareunia, pain on defecation and urination, and infertility [52]. The prevalence of endometriosis in the female population is associated with a significant economic burden on health care systems worldwide. In fact, the expenditures associated with this condition are estimated at approximately USD 22 billion in the United States alone and GBP 12.5 billion in the United Kingdom in treatment, lost work, and health care costs [53]. Of note is that in 2008, the European EndoCost study estimated an average annual expense of EUR 10,000 per patient with endometriosis, highlighting the significant economic burden associated with this condition [53]. In addition, it is important to consider that complications associated with endometriosis are not limited to pain and infertility, as some long-term sequelae can include fibrosis, adhesions, and malignant transformation. Importantly, knowledge about endometriosis has grown significantly in recent years, to the point that it is no longer considered a condition exclusively involving the endometrium, but extending far beyond the pelvic region. Indeed, endometriosis has been reported to affect liver and adipose tissue metabolism, leading to systemic inflammation that alters gene expression in the nervous system, predisposing those with the condition to pain sensitization and mood disorders [54].

Although substantial investigations are needed to clarify the endometriosis etiology, many authors argue that the development of this disorder depends on abnormal growth of the endometrium outside the uterine cavity and involving ovaries, fallopian tubes, and other pelvic organs. The development of endometriotic lesions could be due to retrograde transport of menstrual blood, which is responsible for implantation of endometrial-derived cells within the peritoneal cavity [55,56]. However, during the reproductive age, many women experience retrograde menstruation, but only 10% of them develop endometriosis, suggesting the need for further studies aimed at clarifying endometriosis etiology. Nevertheless, endometrial growth in the abdominal cavity can give rise to superficial peritoneal endometriosis, which accounts for about 80% of the endometriosis cases. However, ovarian endometriosis and deep endometriosis may develop in a proportion of cases. Importantly, these three subtypes of endometriosis are not mutually exclusive, as they can be found at the same time and not only as separate entities. In addition, in some cases the involvement of the recto-sigmoid area of the gastrointestinal tract (intestinal endometriosis), bladder epithelium (bladder endometriosis), or extra-abdominal regions (extra-abdominal endometriosis) [57] may be found.

### 2.7. Vulvodynia

Vulvodynia, with an estimated prevalence in the female population between 8 and 10% [58], is a syndrome characterized by chronic pain around the vulva that occurs both under normal conditions and/or during sexual intercourse. This condition is associated with a huge psychological impact as affected women are prone to dyspareunia, physical disability, anxiety, and depression [59,60]. In addition, chronic pain is the defining symptom of the condition as women with vulvodynia often cannot tolerate even contact with their underwear [61]. Sometimes, such pathology is accompanied by hypertonicity of the pelvic floor muscles as pressure at the level of the anus elevator muscle group triggers allodynia [62]. Importantly, the onset and maintenance of vulvodynia involves muscular and autonomic dysfunction of the pelvic floor, underscoring the importance of interventions aimed at retraining this anatomical area for the well-being of women with such pathology [58].

### 2.8. Pelvic Inflammatory Disease

The term pelvic inflammatory disease (PID) refers to an infection of the upper genital tract due to various pathogens, such as Chlamydia trachomatis or Neisseria gonorrhoeae, affecting 4.4% of sexually active women [63,64]. Specifically, these sexually transmitted pathogens can cause disruption of the normal protective barriers of the lower reproductive tract and travel up the genital tract to the fallopian tubes and, in some cases, to the surrounding pelvic organs. Following the initial infection, it is common to develop polymicrobial infection by bacterial vaginosis-associated pathogens, respiratory pathogens, and enteric pathogens [65]. The resulting inflammatory processes include endometritis, salpingitis, tubo-ovarian abscess, and pelvic peritonitis. Importantly, sometimes PID is asymptomatic, making its diagnosis and treatment difficult. Furthermore, although symptoms may present acutely, the condition can often take a subacute or chronic course, with potential long-term sequelae including chronic pelvic pain, ectopic pregnancy, and infertility [66]. Particularly, although most women respond to antibiotic treatment, it has been estimated that 18% will report infertility, 0.6–2% will experience ectopic pregnancy, and up to 30% will report chronic pelvic pain 3 years after treatment [67]. In addition, the risk of developing long-term sequelae is greatly increased in women with severe fallopian tube damage, recurrent infections, and women with compromised immune systems [68]. Even with timely antibiotic treatment, long-term sequelae of PID can develop, highlighting the need to identify potential strategies that can provide relief to women who do not optimally respond to conventional drug treatment.

### 2.9. Cystitis and Pelvic Organ Prolapse (POP)

Recurrent urinary tract infections, which can affect both the upper and lower urinary tracts, appear to be particularly prevalent in women with pelvic floor disorders [69]. Indeed, estimates report that about 40% of women experience a urinary tract infection in their lifetime and about 27% will experience a recurrent infection in the next 6–12 months [70]. Of these, cystitis is defined as an acute, subacute, or chronic bacterial infection of the bladder, associated with pain and burning during urination, with urine that may be foul-smelling and characterized by a cloudy appearance [71]. The immediate consequences of cystitis can be summarized as worsening urinary frequency or urgency, fever, suprapubic tenderness, costovertebral angle tenderness, and the presence of blood or pus in the urine, while more serious complications can be sexual, bladder, and/or bowel dysfunction [72,73]. Interestingly, some evidence has found an association between cystitis and POP, suggesting the need to pay attention to the risk of recurrent infections [70].

POP is a fairly common condition, the exact prevalence of which remains uncertain as estimates ranging from 3 to 50% have been reported [74,75,76,77]. Not surprisingly, this condition can be a significant obstacle to women’s daily lives, highlighting the need for solutions to counteract both the consequences of the disease and the long-term sequelae of associated urinary infections, such as chronic pelvic pain [78,79,80]. Of note: it has been reported that POP per se is not a risk factor for recurrent urinary infections, but women with POP may have elevated urinary residuals, a factor that predisposes them to urinary tract infections [81].

## 3. Treatment of Painful Disorders of the Pelvic Floor

Depending on the condition, different treatment strategies can be adopted for the management of painful pelvic floor disorders, including pharmacological, non-pharmacological, surgical and alternative therapy approaches. Although there are first-choice treatments, the management approach for these disorders is often multidisciplinary and includes, in most cases, physical therapy aimed at retraining the pelvic floor, highlighting the responsibility of the pelvic musculature in the development of pelvic pain.

The pharmacological treatment of first choice for dysmenorrhea consists of the administration of cyclooxygenase (COX)-inhibiting nonsteroidal anti-inflammatory drugs (NSAIDs) to reduce both the concentration of prostaglandins in menstrual fluid and uterine contractility. Although there is no general agreement on which NSAIDs are most effective in the management of dysmenorrhea, ibuprofen, naproxen, mefenamic acid, and ketoprofen are widely used for the management of this disorder [82,83,84,85]. Alternatively, combined oral contraceptives (estrogen-progestin) are able to decrease menstrual volume and prostaglandin secretion, leading to the reduction in intrauterine pressure and uterine contractility [82,83,86,87,88]. Progestin-only contraceptives, transdermal and vaginal contraceptives, and subcutaneous estrogen-releasing implants can also provide valuable help in the treatment of dysmenorrhea [89,90]. Vitamin E has also been reported to suppress phospholipase A2 (PLA2) and COX activity, reducing prostaglandin production and promoting vasodilation and muscle relaxation [91]. In cases of vitamin B1 deficiency, supplementation can reduce muscle cramps, fatigue and reduced pain tolerance, counteracting dysmenorrhea [91]. Several evidences have pointed out an important role of lifestyle in controlling dysmenorrhea. In particular, a diet low in omega-6 fatty acids and rich in omega-3 fatty acids from beans, seeds, fruits, and vegetables results in reduced production of arachidonic acid and, consequently, prostaglandins and leukotrienes [90]. In general, a healthy lifestyle based on a healthy diet, regular exercise, and cessation of smoking and alcohol is associated with relief of dysmenorrhea [83,92]. Finally, other treatments may be transcutaneous electrical nerve stimulation (TENS), acupuncture, acupressure, and topical heat [90], while in rarer cases, surgery by laparoscopic uterosacral nerve ablation, presacral neurectomy, or hysterectomy may be used [93].

Regarding the management of vaginismus and dyspareunia, the most accepted management approach is multidisciplinary and individualized [94]. In particular, botulinum toxin A injections into the puborectum and pubococcygeum may be used, although supporting evidence is limited [34,95], topical application of anesthetics and corticosteroids [31], as well as tricyclic antidepressants and anticonvulsants. In addition, other strategies may include cognitive-behavioral therapy [96,97], patient education on vulvovaginal and pelvic floor anatomy [98], relaxation of pelvic floor muscles by biofeedback [99], vaginal trainers, and systematic desensitization [97]. In rarer cases, surgical treatment by vestibulectomy may be used [33,100].

Treatment of pudendal neuralgia is often ineffective because it is inadequate. In fact, initial evaluations are often performed by gynecologists, urologists, colorectal surgeons, and pain specialists who undertake therapies based on physical therapy, behavioral modifications, analgesics, nerve blocks, radiofrequency, surgical pudendal decompression, and surgical spinal stimulation [101]. Specifically, the conservative approach based on physical therapy seems to be the treatment of first choice. In this context, therapists who specialize in pelvic floor therapy have several manual techniques that are useful for the release of muscle spasms. Typically, therapy is applied through the vagina, but sometimes it can also be applied through the rectum. In addition, therapists may use electrical stimulation and biofeedback [43]. Pharmacological intervention involves the use of muscle relaxants and neuromodulators, such as gabapentin pregabalin, cyclobenzaprine and tricyclic antidepressants, or local medications [102]. As an alternative to conservative treatment, pudendal nerve block, through injection of local anesthetics or steroids, surgical decompression of the pudendal nerve can be used [43]. Therefore, the multidisciplinary approach seems to be the most widely used, although the opportunity for innovative treatments for the management of this syndrome could provide affected individuals with greater and longer-lasting pain relief, significantly improving their quality of life [103].

Treatment of the perineal injury can be conservative, but this approach can take up to 16 weeks, highlighting the severe discomfort to which the woman is subjected. In this regard, several authors have reported that conservative treatment is associated with less pain than suture surgery, already after the first day of follow-up, and reduction in redness, edema, ecchymosis, after 10 days of follow-up [104]. In this context, available treatment options include far-infrared radiotherapy, capacitive-resistive radiofrequency, pelvic floor muscle training, biofeedback electromyography, cold therapy, as well as the administration of TheresienOl, a natural oil that moisturizes tissue, reduces bacterial infiltration and inflammation, and stimulates tissue healing [105,106,107,108]. On the other hand, some evidence supports the interventional option by suture for shorter healing time and better cosmetic outcome, but there is no common agreement on which procedure is more effective although absorbable sutures are associated with better outcomes [109,110]. In addition, suturing of perineal skin tears has been reported to be associated with increased postpartum pain, with major repercussions on daily life, infant management, and maternal well-being, highlighting the importance of careful clinical assessment of treatment choice [108]. In the context of perineal injuries, drug therapy is aimed at combating pain, mainly through the use of acetaminophen and NSAIDs, stimulating wound healing through topical application of estrogen [111], and preventing infection through antibiotic therapy [112].

For the treatment of chronic constipation, several trials support the therapeutic efficacy of pelvic floor retraining using biofeedback therapy, which has been found to be effective in 80% of patients with chronic constipation [113,114]. Alternatively, lifestyle correction aimed at increasing water and fiber intake along with regular exercise may have beneficial effects on bowel motility. However, a pharmacological therapeutic approach is often necessary, based on the use of laxatives to improve intestinal transit or secretagogues, such as lubiprostone and linaclotide, to increase intestinal secretion [50]. Unfortunately, in some cases it is necessary to resort to colectomy surgery, which, although it may benefit some patients, is often associated with substantial short- and long-term morbidity, highlighting the need to experiment with innovative treatments for the resolution of chronic constipation [115].

The management of endometriosis aims to reduce pain and minimize the risk of infertility. In this context, the pharmacological approach, based on the use of contraceptives and/or gonadotropin-releasing hormone (GnRH) agonists to induce suppression of ovulation and menstruation, can result in improvement of algic symptoms. Eradication of lesions appears to reduce infertility but is associated with high recurrence rates [116]. In addition, positive results have been obtained in the treatment of lesions in randomized controlled trials evaluating the efficacy of aromatase inhibitors [117,118], selective estrogen receptor modulators [119], anti-inflammatory agents [120], and immunomodulators [121]. Finally, in recent times, diet-based interventions have been associated with improved symptoms of endometriosis, although the underlying mechanism has not yet been elucidated [122]. In addition, it has been reported that pelvic floor physiotherapy interventions can promote significant functional improvement in women with endometriosis [123], although more recent findings have questioned the usefulness of this strategy in improving urinary, bowel, and bowel function [124]. Therefore, as the resolution of this disease is still far off, the role of alternative interventions aimed at improving symptoms needs to be further studied to improve the quality of life of women forced to live with endometriosis.

Relative to vulvodynia, the absence of a known etiology makes the identification of effective therapeutic approaches for the management and treatment of this condition extremely complicated. In fact, the most widely used treatments are mainly aimed at reducing allodynia and dyspareunia and include corticosteroids, lidocaine, gabapentin, ketamine, as well as hormonal treatments [125]. However, the management of the patient with vulvodynia must necessarily also include emotional and psychological support, as this chronic pain condition dramatically impairs psychological and physical well-being [126]. Interestingly, women with vulvodynia are characterized by hypertonicity of the pelvic floor muscles and poor strength and control of this muscle group [127]. In this regard, manual pelvic floor physical therapy, such as stretching and massage, has been proposed as a potential nonpharmacological strategy that can facilitate muscle relaxation and circulation [128]. Alternatively, acupuncture and intravaginal TENS have also been proposed as potential nonpharmacological strategies that can alleviate the symptoms of vulodynia [129,130]. Pharmacologic treatment includes application to the vulvar vestibule of 5% lidocaine overnight cream [131], also in combination with oral desipramine treatments [132]. More invasive pharmacological treatments include the administration of botulinum toxin type A and subcutaneous injections of enoxaparin sodium [133,134]. Finally, treatments that can be adopted in the treatment of vulvodynia are vaginal dilators, cognitive behavioral therapy, biofeedback, hypnotherapy, laser therapy, cold knife vestibulectomy, and estradiol/testosterone creams, gabapentin, ketamine, and amitriptyline [61]. Therefore, intervening on the tonicity of the pelvic floor muscles could be a promising management strategy for vulvodynia that can reduce pain and improve the quality of life of affected women.

In the case of pelvic inflammatory disease, antibiotic treatment can be administered empirically, orally, and on an outpatient basis and usually involves cephalosporin, doxycycline, or metronidazole and is also recommended for sexual partners. However, in cases of pregnancy, failure of oral therapy, nausea, and fever, hospitalization of the patient with parenterally administered treatment may be necessary, with transition to oral treatment only after 24 h of clinical improvement [64].

Relative to POP, the most widely accepted treatment strategy is pelvic floor physiotherapy, although the use of vaginal devices capable of supporting prolapsed organs or surgery may also be employed [135]. Alternatively, a hypopressive breathing technique can be used, which involves reducing intra-abdominal pressure and, consequently, reflex activity of the abdominal wall and pelvic floor muscles [136]. Nevertheless, Resende et al. conducted a randomized controlled trial (RCT) to compare the effectiveness of hypopressive exercises with pelvic floor muscle training and found better results, in terms of vaginal bulging, heaviness or dragging in the lower abdomen, and stress incontinence in the group undergoing pelvic muscle training [137]. In cases in which POP predisposes to urinary tract infections and cystitis, treatment with beta-lactam antibiotics is recommended [71], although the use of nonantibiotic treatments including urine alkalinization, cranberry products, probiotics, NSAIDs, D-mannose, phytotherapy, methenamine hippurate, oral immunostimulants (immunotherapy), topical estrogens, vitamins, and acupuncture has also been documented [138]. Finally, surgery includes several procedures such as sacral colpopexy, sacrospinous colpopexy, uterosacral colpopexy, and transvaginal mesh [139].

Table 1 summarizes the main treatment approaches for painful pelvic floor conditions.

## 4. CBD and the Endocannabinoid System (ECS)

In recent years, evidence on the pharmacological efficacy of compounds isolated from *Cannabis sativa* has increased significantly, to the point that several countries have allowed the use of oils or extracts for therapeutic purposes [140]. Specifically, phytocannabinoids constitute a group of bioactive molecules derived from cannabigerolic acid (CBGA) that are present in high concentrations in the floral portion of *Cannabis sativa*, but can also be found in other plant species, albeit in smaller amounts [141]. Among these, Δ9-Tetrahydrocannabinol (Δ9-THC) and CBD have been identified as the compounds with the most obvious biological activity as Δ9-THC is known for appetite stimulation and psychoactive effects, while CBD has mainly anti-convulsant and anti-inflammatory properties [142,143,144]. Importantly, more than 500 compounds have been identified in *Cannabis sativa* including 125 cannabinoids, 42 non-cannabinoid phenolic compounds, 34 flavonoids, 120 terpenes, and at least two alkaloids, making scientific research on the therapeutic properties of this plant very complicated [145]. Although some cannabinoids have raised research interest for their therapeutic properties, the complexity of the chemical profile of *Cannabis sativa* requires extensive study aimed at understanding the synergistic action of all the compounds in the plant, a characteristic referred to as the “entourage effect” [146]. Nonetheless, CBD has captured the attention of physicians for its versatility and reduced risk of addiction, suggesting its potential application in several disease contexts [147].

The polypharmacological effects and the absence of a marked psychoactive effect have made CBD an ideal candidate for the management and treatment of several conditions, such as pain and anxiety, eliminating the risk associated with the typical consequences of substances of abuse [148,149]. In fact, CBD is able to activate different molecular pathways in numerous tissues where, through interaction with specific receptors, it can play the role of agonist, antagonist, and positive or negative allosteric modulator [150]. Of note: the study of the effects of CBD and Δ9-THC led to the discovery of a complex molecular/biological system, distributed throughout the body, known as the endocannabinoid system (ECS). This system consists of specific receptors, called cannabinoid receptors (CBRs), compounds of a lipid nature that serve as endogenous ligands for CBRs, and the mechanisms involved in the synthesis, transport, and degradation of these ligands [151]. The most important endogenous ligands of the ECS, or endocannabinoids, are N-arachidonoyl ethanolamine (AEA), also known as anandamide, and 2-arachidonoylglycerol (2-AG), although other endocannabinoids whose biological relevance has not yet been fully elucidated [152] have been identified. Endocannabinoids, whose synthesis and degradation are under the control of different enzymatic pathways, interact with different types of CBRs, such as G-protein-coupled receptors, represented by cannabinoid type 1 (CB_1_R) and type 2 (CB_2_R) receptors, ligand-dependent ion channels, such as transient receptor potential vanilloid 1 (TRPV1), as well as nuclear receptors, such as peroxisome proliferator-activated receptors (PPARs) [153,154]. This sophisticated system is involved in the regulation of a plethora of physiological processes, thus representing a potential therapeutic target for mood and anxiety disorders, pain management, cardiovascular disorders, and many other pathological conditions [155].

Interestingly, numerous evidences have shown that CBD is able to regulate skeletal muscle metabolism, to the point that it has been suggested as an innovative strategy for the control of muscle spasticity in various pathological conditions [156,157]. The importance of this remarkable property of CBD in the context of painful pelvic floor conditions lies in the fact that this group of disorders is often caused by altered tonicity of pelvic floor muscles. Although the mechanism of action underlying CBD-induced regulation of muscle metabolism has not yet been fully elucidated, Ghovanloo and colleagues have demonstrated the involvement of voltage-dependent sodium channels (Nav). Specifically, the authors demonstrated that CBD can inhibit Nav1.4 activity by binding within the pore and modulating skeletal muscle membrane elasticity [158]. The Nav family of channels is abundantly distributed throughout the body and play a crucial function in the context of nervous system excitability, regulating pain, seizures, muscle problems, arrhythmias, and more [159]. Of note, pharmacological modulation of Navs can produce positive results in the context of vaginal hyperalgesia associated with endometriosis and vulvodynia, highlighting the importance of further knowledge regarding the inhibitory role of CBD in Nav channels [160,161]. Nonetheless, although Nav modulation represents an interesting, innovative mechanism worthy of further study, the role of the endocannabinoid system in pelvic pain control is well known and documented [162]. In fact, TRPV1 overexpression has been verified in the peritoneum of women with chronic pelvic pain, suggesting a role of this receptor in the onset and maintenance of algic symptoms [163]. In addition, Asfaw et al. demonstrated that TRPV1 activation in a rat model of pelvic organ cross-sensitization can result in detrusor muscle instability, highlighting both the role of TRPV1 in pelvic pain and the existence of a reciprocal influence between pelvic floor-associated structures [164]. In this context, TRPV1 expression was found in the gastrointestinal tract, genitourinary system, and smooth and skeletal muscles, highlighting its importance in pelvic homeostasis [165,166,167,168].

Regarding CB_1_R expression, it has been reported that these receptors are abundantly represented in skeletal muscle at the level of the cell membrane, although a subpopulation is also present in mitochondria, where they play a key role in morphology and function [169]. Indeed, treatment of a rat muscle cell line with Arachidonyl-2ʹ-chloroethylamide, a selective CB_1_R agonist, promoted IL-6 gene expression, suggesting a role for this receptor in inflammation and endocrine function in skeletal muscle [170]. Therefore, modulation of CB1R activity may be a promising target for treating conditions of muscle atrophy through regulation of muscle metabolism and regenerative capacity [171]. Similarly, CB_2_R has also been reported as a promising therapeutic target for the management of muscle fibrosis, as it is involved in the regeneration processes of damaged skeletal muscle [172]. Of note, although CB_2_R is expressed in several organs and tissues, it would appear to be particularly represented in the immune system, suggesting a key role in inflammation and immune responses [173]. Taken together, this evidence shows a crucial role of CBRs in skeletal muscle homeostasis, suggesting their potential involvement in painful pelvic floor conditions associated with altered muscle tone. Importantly, the endocannabinoid anandamide and 2-AG are produced by skeletal muscle and regulate acute neuromuscular junction transmission, limiting the efficiency of excitation–contraction coupling [174]. In other words, the structure, function, and homeostasis of entire apparatuses, such as the muscular, endocrine, nervous, and immune systems, are closely dependent on the endocannabinoid system neurotransmission [175]. Alteration of any of the parts involved in this complex system can result in an imbalance of optimal muscle function, leading to disorders affecting extensive anatomical areas [176,177,178,179]. Moreover, in addition to skeletal muscles, the endocannabinoid system regulates the activity and function of other pelvic organs and tissues, highlighting the importance of this system in the well-being and optimal interconnection of the structures that make up the pelvis [180,181,182].

**Figure 1:**
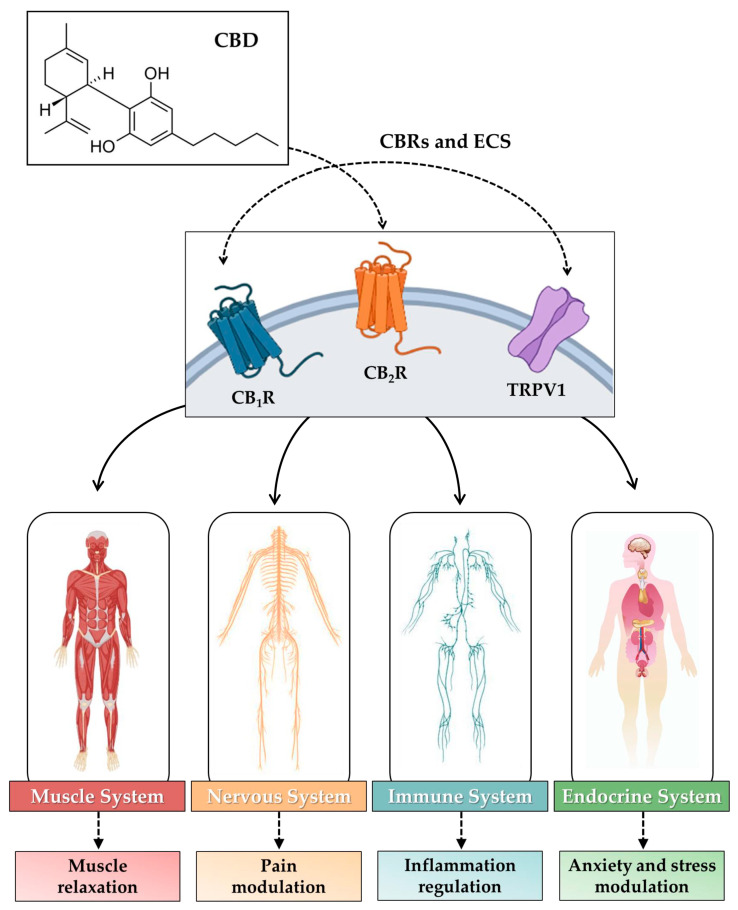
Role of the endocannabinoid system (ECS) in the muscle, nervous, immune, and endocrine systems. The activation of cannabinoid subtype 1 receptor (CB_1_R), cannabinoid subtype 2 receptor (CB_2_R), and transient receptor potential vanilloid 1 (TRPV1) in the muscle, nervous, immune, and endocrine systems by cannabidiol (CBD) promotes muscle relaxation, pain reduction, regulates inflammatory responses, and modulates anxiety and stress.

Figure 1 is a schematic representation of the effects of endocannabinoid stimulation by CBD in muscle, nerve, immune, and endocrine systems.

## 5. CBD as a Treatment Strategy for Painful Pelvic Floor Conditions

CBD’s anti-inflammatory and analgesic properties and ability to interact with CBRs suggest its potential role in the management of painful pelvic floor conditions by promoting vaginal wellness and improving pelvic function. Unfortunately, evidence supporting the efficacy of CBD in counteracting pain symptoms is still limited, highlighting the need to conduct clinical trials aimed at confirming the role of the phytocannabinoid in pelvic wellness. Nevertheless, some authors have reported significant results supporting the therapeutic effect CBD in pelvic floor treatment, which may suggest its role as an integrative therapy. In this context, Sinclair and colleagues conducted a study to investigate opinion on the use of medicinal cannabis in a population of Australian women with primary dysmenorrhea. The authors found considerable interest in women regarding the use of cannabis-derived products for the management of such painful pelvic disorders, highlighting the need to conduct clinical trials aimed at verifying their efficacy and safety [183]. It is possible that stimulation of CBRs by CBD may limit calcium influx and uterine stimulation by counteracting dysmenorrhea, as observed by Peng et al. in a mouse model of the condition [184]. Growing interest by several companies in the potential of CBD in the management of dysmenorrhea has led to the production of a tampon coated with CBD oil, although the efficacy, safety, and mechanism of action of such a product have not been tested [185]. Although the evidence regarding the efficacy of CBD in controlling dysmenorrhea is promising, the absence of high-quality evidence limits its use, highlighting the need to conduct studies to verify its efficacy and safety. In fact, most of the evidence regarding the efficacy of CBD in painful pelvic floor conditions relates to endometriosis and vulvodynia. In this context, because pain is the predominant symptom, CBD’s analgesic properties have attracted great interest in the treatment of these conditions. Indeed, in 2020 Sinclair and colleagues published the results of a survey of Australian women with endometriosis aged 18 to 45 years. Responses analyzed by the authors showed that 76% of women with endometriosis use self-management strategies to achieve pain relief. Specifically, 13% of these used medicinal cannabis for symptom management, reporting reduced pain, reduced medication intake, and improved sleep, with infrequent side effects, highlighting the need for further evidence to confirm the efficacy and safety of CBD in managing endometriosis symptoms [186,187]. The molecular mechanism underlying the beneficial effects of CBD in endometriosis was partly elucidated by Genovese et al., who experimentally induced endometriosis in 8- to 10-week-old Sprague-Dawley rats treated with oral CBD at a dose of 10 mg/Kg. Of note: the authors observed a volumetric reduction in lesion area in CBD-treated rats compared with untreated rats, suggesting the ability of phytocannabinol to counteract the development of endometriotic lesions. In addition, CBD treatment reduced lipid peroxidation and the expression of nicotinamide adenine dinucleotide phosphate (NADPH) oxidases 1 and 4 (Nox-1, Nox-4), and enhanced the expression of superoxide dismutase and glutathione levels, highlighting its antioxidant power. Finally, CBD counteracted inflammation by inducing down-regulation of matrix metallopeptidase-9 (MMP-9), inducible nitric oxide synthase (iNOS), and transforming growth factor-β (TGF-β) [188]. These results were confirmed by Okten and colleagues, who found a reduction in the levels of interleukin-6 (IL-6), tumor necrosis factor α (TNF-α), and vascular endothelial growth factor (VEGF) in blood and peritoneal fluid in female Wistar albino rats with experimentally induced endometriosis, concomitantly with an improvement in oxidative status [189]. Taken together, this evidence demonstrates the extraordinary anti-inflammatory, antioxidant, and antiangiogenic power of CBD, setting the stage for thoroughly investigating the mechanism of action, efficacy, and safety of phytocannabinol in the female population.

CBD’s ability to modulate pain by acting not only on the inflammatory component but also on the neuropathic component makes it an ideal candidate for the management of vulvodynia [190]. In fact, Barach et al. reported the results of an online survey involving 38 women with dyspareunia associated with vulvodynia who resorted to medicinal cannabis for symptom management. The authors reported that cannabis use was associated with improvement in vulvodynia symptoms, although women’s expectations were higher regarding the relief of acute, prickly symptoms, suggesting the need to conduct RCTs to evaluate the actual efficacy and potential placebo effect of medicinal cannabis [191]. In addition, in 2022 Liang et al. conducted a systematic literature search with the aim of evaluating the analgesic efficacy of medicinal cannabis in gynecological pain. The authors included 16 articles in the review including women with pelvic pain associated with different conditions such as endometriosis chronic pelvic pain, vulvodynia, endometriosis, interstitial cystitis, or cancer. Of note, 61 to 95.5% of women reported a reduction in gynecological pain. In fact, all prospective cohort studies evaluated and one RCT analyzing the efficacy of drugs combined with palmitoylethanolamide (PEA), a fatty acid amide that potentiates endogenous cannabinoids, reported significant pain reduction after 3 months of treatment [16]. Thus, evidence in the literature supports the efficacy of cannabis-derived products, and CBD in particular, in reducing pelvic pain in a variety of conditions, suggesting its potential role in the management and treatment of painful pelvic floor conditions. Unfortunately, the evidence supporting CBD is currently still limited, and interpretation of the available results requires caution due to the different routes of administration, dosages, and formulations of medicinal cannabis that do not allow for ideal comparisons between published data. Furthermore, it is important to note that despite the analgesic and anti-inflammatory properties of CBD, the beneficial effects found are mainly related to symptom improvement. In fact, Irving and colleagues conducted an RCT evaluating the efficacy of CBD capsules in patients with ulcerative colitis and found no statistically significant clinical remission of the condition compared with the placebo-treated group [192]. Similarly, Naftlai et al. reported that treating patients with Crohn’s disease with a low dose of CBD, although found to be safe, did not result in beneficial effects, probably due to low dosage or lack of synergy with other cannabinoids [193]. However, these studies did not investigate the efficacy of CBD in improving algic symptoms, necessitating a more thorough evaluation of the therapeutic importance of CBD in pain conditions. In this context, Frane et al. investigated the effects of CBD in patients with osteoarthritis, reporting an association between cannabinoid use and improvements in pain, physical function, and sleep quality [194]. Therefore, although CBD does not intervene in pathophysiological mechanisms, its ability to modulate pain makes it a potential candidate for the management of painful pelvic floor conditions.

It is noteworthy that, in addition to CBD, other cannabinoids have also been considered for analgesic effects in the context of painful pelvic floor conditions. Particularly, repeated exposure to THC in a mouse model of surgically induced endometriosis is known to alleviate mechanical hypersensitivity, suggesting its potential role in the management of pain characterizing pelvic floor disorders [195]. In addition, the involvement of other cannabinoids in neuropathic pain control, such as cannabinol and cannabigerol, has been suggested, although evidence for their efficacy in painful pelvic floor conditions is still limited [196].

## 6. Limits of the Study

Some limitations of our review are related to the heterogeneity of studies addressing the use of CBD in painful pelvic floor conditions. First, the mode and frequency of administration can vary considerably, making it difficult to appropriately compare studies. Indeed, the use of CBD for the self-management of chronic pelvic pain may involve daily, weekly, or monthly intake, complicating the quantification of CBD taken by patients with painful pelvic floor disorders [197]. In addition, most RCTs aimed at evaluating the efficacy of CBD on painful pelvic floor conditions are characterized by a low number of enrolled participants. This sample limitation could depend on both the difficulty of externalizing one’s pathological condition and the preconceptions associated with cannabis use. Moreover, because some authors report some side effects related to CBD use, especially drowsiness and gastrointestinal symptoms, further studies are needed to determine the safety of CBD. Therefore, it is necessary to conduct RCTs with a large number of participants and with a long follow-up in order to evaluate both the long-term efficacy of CBD in pelvic pain and the side effects in terms of cognitive function and ability to perform activities of daily living.

## 7. Conclusions

Disorders affecting the pelvic floor are associated with a significant reduction in quality of life, as this anatomical region is involved in numerous physiological functions whose alteration can have a major negative impact on the lives of individuals. Particularly, these disorders are remarkably prevalent in the female population, with major repercussions in daily life, especially in relationships with partners. In fact, gynecological and pelvic pain emerges as the common symptom of painful pelvic floor conditions, the management of which requires special attention because of the emotional, psychological, and relationship repercussions it entails. Stimulation of the endocannabinoid system with CBD may result in an improvement in the typical symptomatology of these disorders, promoting the well-being of this complex and delicate anatomical area. Indeed, as suggested by evidence in the literature, CBD’s analgesic, anti-inflammatory, and antioxidant properties make it an ideal candidate for the management of pelvic disorders. However, the mechanism by which CBD acts on the endocannabinoid system by reducing vaginal pain needs important clarification. In addition, there is a need to conduct quality clinical trials aimed at determining the efficacy, harmlessness, and potential placebo effect of CBD. The ability to use CBD safely, including in combination with currently used therapies, could promote clinically meaningful outcomes and improve the quality of life for women with painful pelvic floor disorders.

## Figures and Tables

**Table 1:** A schematic representation of the main treatment approaches for painful pelvic floor conditions.

Painful Pelvic Floor Conditions	Pharmacological Treatment	Non-Pharmacological Treatment	Surgical Treatment
**Dysmenorrhea**	- NSAIDs (ibuprofen, naproxen, mefenamic acid, ketoprofen) - Oral contraceptives (estrogen-progestin, progestin-based) - Transdermal and vaginal contraceptives - Estrogen-releasing subcutaneous implants	- Vitamin E - Vitamin B1 - Diet correction - Exercise - Cessation of smoking and alcohol - TENS - Acupuncture - Acupressure - Topical heat	- Laparoscopic ablation of the uterosacral nerve - Presacral neurectomy - Hysterectomy
**Vaginismus and dyspareunia**	- Botulinum toxin A injections - Topical application of anesthetics and corticosteroids - Tricyclic antidepressants - Anticonvulsants	- Cognitive-behavioral therapy - Patient education on vulvovaginal and pelvic floor anatomy - Biofeedback for relaxation of pelvic floor muscles - Vaginal trainers - Systematic desensitization	- Vestibulectomy
**Pudendal nerve neuralgia**	- Muscle relaxants - Neuromodulators - Local drugs - Blockage of the pudendal nerve by injection of local anesthetics or steroids	- Pelvic floor therapy for muscle spasm release - Electrical stimulation - Biofeedback	- Surgical decompression of the pudendal
**Perineal trauma**	- Paracetamol - NSAIDS - Topical estrogens - Antibiotic therapy	- Far infrared radiotherapy - Capacitive-resistive radiofrequency - Pelvic floor muscle training - Electromyography biofeedback - Cryotherapy - TheresienOl	- Suture surgery
**Chronic constipation**	- Laxatives for intestinal transit - Secretagogues (lubiprostone, linaclotide)	- Increased intake of water and fiber - Exercise - Biofeedback for pelvic floor reeducation	- Colectomy
**Endometriosis**	- Contraceptives - Gonadotropin-releasing hormone agonists - Aromatase inhibitors - Selective estrogen receptor modulators - Anti-inflammatory agents - Immunomodulators	- Diet correction - Pelvic floor physiotherapy	- Eradication of pelvic lesions
**Vulvodynia**	- Corticosteroids - 5% lidocaine cream - Estradiol/testosterone creams - Gabapentin-based creams - Ketamine-based creams - Amitriptyline-based creams - Oral desipramine - Botulinum toxin type A - Subcutaneous injections of enoxaparin sodium - Hormonal treatments	- Pelvic floor manual physical therapy - Agopuncture - Intravaginal TENS - Vaginal dilators - Cognitive behavioral therapy - Biofeedback - Ipnotherapy - Laser therapy	- Cold knife vestibulectomy
**Pelvic inflammatory disease**	- Antibiotic therapy (cephalosporin, doxycycline or metronidazole)	/	/
**Pelvic Organ Prolapse**	/	- Pelvic floor physiotherapy - Vaginal devices for prolapsed organ support - Hypopressive breathing - Pelvic floor muscle training	- Sacral colpopexy - Sacrospinous colpopexy - Uterosacral colpopexy - Transvaginal mesh
**Cystitis**	- Beta-lactam antibiotics - NSAIDS - Oral immunostimulants - Topical estrogens	- Alkalinization of urine - Cranberry products - Probiotics - Vitamins - Phytotherapy - Acupuncture	/

NSAIDs: nonsteroidal anti-inflammatory drugs; TENS: transcutaneous electrical nerve stimulation.

## Data Availability

No new data were created or analyzed in this study. Data sharing is not applicable to this article.
